# Factors contributing to men’s reluctance to seek HIV counselling and testing at Primary Health Care facilities in Vhembe District of South Africa

**DOI:** 10.4102/phcfm.v8i2.996

**Published:** 2016-05-31

**Authors:** Pfungwa Mambanga, Robert N. Sirwali, Takalani Tshitangano

**Affiliations:** 1 Department of Public Health, University of Venda, South Africa

## Abstract

**Background:**

Voluntary HIV antibody Counselling and Testing (HCT) is a cornerstone of HIV prevention in South Africa because it has the potential to prevent HIV transmission. The government of South Africa has for a long time been investing heavily in fighting the spread of HIV and/or AIDS. However, men rarely utilise this service.

**Aim:**

The aim of this study was to explore the factors contributing to the reluctance of men to seek HCT in the primary health facilities in Vhembe District.

**Setting:**

The study was conducted at Vhembe District health offices in Limpopo, South Africa.

**Methods:**

A qualitative research design, anchored on semi-structured interviews as a method of data collection, was used. Fifteen men working at Vhembe health offices were purposively sampled. Data were analysed using the TECHS’s 8 steps method. The approval from Polokwane Provincial offices was guaranteed with participants being protected and respected throughout the study.

**Results:**

The response rate per question was 100% with all 15 participants willing to answer all the raised questions, though with different views and opinions. The majority of the interviewees indicated that they were aware of HCT services. Stigma as a societal reaction to disease, governmental policies, and attitudinal factors made men refrain from seeking counselling and testing from public health facilities.

**Conclusion:**

There was a high level of HCT awareness among men in Vhembe District. However, attitudinal and political barriers, stigma, and cultural practices such as circumcision were cited as the reasons for the low level utilisation of HCT services.

## Introduction

South Africa (SA) has 0.7% of the world’s population but accounts for 17.0% of the global HIV infections, and carries a quarter of sub-Saharan Africa’s total burden of Human Immunodeficiency Virus.^1,2,3^ SA has the highest number of people living with HIV in the world, with an estimated 5.3 million people living with HIV and AIDS.^1^ The epidemic in SA has stabilised at around 11%, with the rates of new infection starting to decline, particularly among young people. SA’s third national HIV Prevalence, Incidence, and Communication Survey conducted in 2008 found that HIV prevalence has declined among children aged 2–14, from 5.6% in 2002 to 2.5% in 2008. A decline in new infections has also been noted among teenagers aged 15–19.^4^ Furthermore, the incidence declined by 35.0% in the 15–49 age group between 2002 and 2008.^3^

Although the first signs of the decline in HIV incidence have been noted in SA, the national prevalence rate, and in particular prevalence rates among some age cohorts, remains unacceptably high. In fact, SA is considered a hyper-endemic country with prevalence among 15–49 year olds being above 15%. Furthermore, the burden of HIV is significant, the death rate among young adults has doubled in the past decade, and nearly 2 million children and teenagers have been orphaned.^3^ Nationwide, across provinces and districts in SA targets were set for the HIV Counselling and Testing (HCT) programme. Each of the facilities, including hospitals and clinics, has a set target. The objectives of the campaign were to mobilise people to know their status; reinforce prevention messages; increase HIV health seeking-behaviour; and increase access to treatment, care, and support. The benefits of knowing one’s status are that one can plan the future and learn how to protect oneself and others; and that one can access care and support, including treatment to prevent opportunistic infections and treatment of an illness like TB.^3^

The reason for initiating the HCT campaign was based on the magnitude of the epidemic in SA and the Department of Health (DOH) has developed a 10-point plan as a strategy for the government to enhance service delivery.^2^ Among others, the issue of accelerating the implementation of the HIV and AIDS strategic plan and increased focus on TB and other communicable diseases was identified. Furthermore, it was meant to involve men in the fight against HIV and AIDS.^4^ As people grow to be adults it generally brings privileges to the nation; however, it carries high health costs. Older men frequently delay seeking health care for illnesses that could be prevented or cured.^5^ With 10.9% people getting tested every day, HCT presents an entry point for prevention and treatment, care and support, and as a vehicle for social mobilisation for HIVWorldwide.^3^ The rationale of the study is that men have a vital role to play in curbing the spread of HIV and in mitigating its impact. From a historical perspective, men are still accorded a much higher status by the society and are relied upon to make decisions that have a bearing on how the society conducts itself. Therefore, in as much as men continued to resist going for HCT, the war against HIV and AIDS cannot be won because most men regard women as second-class citizens and still treat them badly.^2^

### Purpose of the study

This article aims to investigate the factors that contribute to men’s reluctance to seek HCT at primary health care facilities in Vhembe District, SA.

### Objectives of the study

The objectives of this study were as follows:

- Explore the level of awareness of men regarding HCT services.
- Explore the attitudes of men towards HCT services.
- Explore the socio-cultural and political factors that prevented men from seeking HCT services.
- Explore the gender and stigma-related factors that influenced men’s level of utilisation of HCT services.

## Research methods and design

### Study design

A qualitative approach method was used to investigate the factors that contribute to men’s reluctance to utilise HCT services. The design was chosen because it gives the participants a formal platform of expressing freely their feelings, attitudes, level of awareness, socio-cultural, political, administrative and legislative factors about the reluctance to HCT at Primary Health Care.^6^ The design was suitable for the study because the researcher was part of the research as observer and interviewer, and served as interpreter of some inquiry. The researcher is therefore deeply immersed with the data.^7^

### Setting

The study was conducted in Vhembe District Department of Health, which is situated at the northern part of government complex, Thohoyandou, Mphephu Street, next to Khoroni tusk sun. The Department has several offices with the following divisions, namely finance, human resources, primary health care, health care support, hospital services, national health insurance, and health special programme.

### Population and sampling strategy

Men who were working in Vhembe District Department of Health irrespective of years, experience, and years at work, constituted the study population. Non-probability, purposive sampling method was used to select 15 men.^8^

### Data collection tool

An interview guide was developed based on the objectives of the study. The central question was, ‘what do you think are factors that contribute to the reluctance of men in utilising HCT at primary health care facilities’. The interview guide contained open-ended questions, which covered all the objectives and/or variables.

### Data collection method and process

The semi-structured interview method was used for data collection. In this method, each of the 15 men was individually interviewed. The questions in the guide were open-ended, which made it possible to probe further once a respondent provided an answer to each one of them. Thus, the interview was a conversation in which the responses guided the direction of the interviews.^8^ The period of the interview took about 40 min to 1 h for each participant. With the consent of the respondents, data was collected using a voice recorder with notes taken during the interview to record observed non-verbal communications. Appointments were made with each of the participants a week before to avoid inconveniences. Participants determined the venue of the interview and the researcher made necessary arrangements to visit each participant at their homes or any identified place. Logistics were made to avoid any disturbances during the interview such as booking a free room.

### Data analysis

Data were analysed thematically (for the content of the interviews) using the Tesch’s 8 Steps method.^6^ Lincoln and Guba’s model comprised of four criteria to measure trustworthiness of the findings namely credibility, dependability, applicability, and neutrality was adopted.^9^

### Trustworthiness

Trustworthiness refers to the extent to which a research is worth giving attention to and worth taking note of. To ensure credibility in this study, the researcher used interviews and remained in the field for a prolonged engagement with the participants. The other aspect that the researcher did to ensure credibility was the use of a variety of sources in data collection using more than one tool. This included the use of a tape recorder and field notes.

Dependability involves appropriateness of inquiry, decision, and methodological changes. The process was reviewed constantly to ensure that inquirer bias did not influence the inquiry. Dependability was achieved by describing the research method fully and by describing the research protocol with the research team and the independent coder. A tape recorder was used to increase reliability when doing all the interviews. The proposal of this study was presented to a panel of University of Venda Higher Degrees Committee experts that was comprised of research professors and doctors, and the study was awarded the ethical clearance with a belief that the methodology was dependable. As an experienced health professional, the researcher’s knowledge of HCT added value to the process of data collection, coding, formulation, and naming of sub-themes which then attest to the dependability of the results (Table 1).

**TABLE 1:** The Themes and sub-themes of the study.

Themes	Sub-themes	Questions
1. Level of awareness of the participants	Men are aware of HCT services Respondents agreed on the availability of HCT facilities Respondents have the knowledge of HCT service.	Have ever heard of HCT? What is HCT, and where can you access the services?
2. Attitudes of participants towards HCT	Majority of the participants have negative attitudes towards HCT services. Men are afraid of being tested positive. Men have pride hence they feel that to be tested is an embarrassment. Respondents accuse the behaviour of counsellors as a catalyst of negative attitude towards HCT services.	Have you ever been tested? If no, what was your reason/s?
3. Socio-cultural factors that prevent men from seeking HCT services	Men believe in cultural practices rather than the medical and professional practices. Men view HCT as there for single people not for the married.	Apart from HCT are there any other social or cultural practices who engage in? Do you think HCT is marital status based?
4. Political, administrative and legislative factors that prevent men in seeking HCT services	Men expressed that politics is failing to relay and cooperate the medical and traditional health practitioners for a common goal. Men view administrative personnel and labour laws as hindrances to HCT service utilisation.	1. What the political, administrative and legislative barriers to HCT?
5. Gender and stigma-related factors that influence men in seeking HCT services	Gender and stigma-related issues have made many men reluctant to utilise HCT services. Participants identified ways of enabling men to utilise HCT services.	Are there gender and related issues with the scope of HCT objectives? Are there solutions to all these contributing factors?

*Source*: Authors own work

HCT, HIV Counselling and Testing.

The researcher ensured confirmability by making use of an independent coder. The incorporation of an audit procedure in which the researcher clearly described each stage of the research process, exploring and justifying what was done and presenting reasons for the decision taken. For transferability the researcher asked another experienced researcher to read two randomly selected transcripts and to identify major categories. These were compared with the researcher’s own coding.

### Ethical considerations

Permission for data collection was obtained from the higher degree committee of the School of Health Sciences of the University of Venda (project number SHS/14PH/12/12/1508). The provincial health department gave the letter for data collection at Vhembe district. Once approval from the ethics committee of the university and the Department of Health Limpopo Province was obtained, each respondent was given specific information regarding participation in the study. Explanation was done at the level of each respondent’s understanding. After the researcher was satisfied that the respondent understood his rights and responsibilities in the study, each respondent was requested to give a written informed consent. Ethical principles of justice, beneficence, fairness, anonymity, confidentiality, and self-determination were observed when dealing with participants. The interview guide of respondents was given codes of numbers (1, 2, 3….) to protect the identity of the interviewee.

### Limitations of the study

Although the research findings of this study can be crucial for comparison on the factors contributing to men’s reluctance to HCT services across SA, the small sample size and setting of the study pose a limitation for generalisation. A further limitation is that the participants may have been biased in expressing exact factors behind their reluctance in seeking HCT services because the interviewer was once the director at the health offices in Vhembe.

## Results

Underneath is a detailed interpretation and presentation of the identified themes and sub-themes with regard to factors that contribute to men’s reluctance to seek HCT at primary health care facilities of Vhembe District. The responding rate per question was 100% (*n* = 15).

### Theme 1: The level of awareness of the participants regarding HIV Counselling and Testing services

#### Sub-theme 1: Men are aware of HIV Counselling and Testing services

Twelve of the 15 participants indicated that they were absolutely aware of HCT services. One of them confirmed his awareness by saying:

‘I know about HCT services, rendering testing for HIV at hospitals, clinics and even where I work they also tests HIV. Sometimes at taxi ranks they come with their mobile cars for testing.’ (Participant 2)

Although two other men responded positively to the question, they had a shallow understanding of HCT services. The remaining interviewee confirmed that:

‘ HCT is it that we will be talking of HIV/AIDS?’ (Participant 5)

#### Sub-theme 2: Respondents confirmed the availability of HIV Counselling and Testing facilities

All the participants appreciated the availability of HCT services in Vhembe district. One of the men said:

‘I know HCT is done in all public health institutions, private facilities even at places like Vhembe District.’ (Participant 7)

Another one concurred that:

‘When you go to universities and colleges they have small clinics where they offer HCT for students.’ (Participant 4)

#### Sub-theme 3: Respondents have the knowledge of HIV Counselling and Testing services

The respondents have shown the prerequisite knowledge about the meaning of HCT services. They actually verbalised that counsellors are responsible individuals in explaining the scope and meaning of HCT services. One of the majority participants confirmed that:

‘We sometime attends workshops of HIV counseling and testing at our workplace and counselors are the ones handling all this especially giving guidance.’

However, the minority (*n* = 3) of them indicated that they do not really understand the meaning of HCT.

One of the participants indicated that some people have false information about HCT as the participant said:

‘I know HCT that it gives people ARVs treatment and when people improve they look beautiful and that spread disease, so I think this program it is there to promote HIV spreading.’

### Theme 2: The attitude of the men towards HIV Counselling and Testing services

#### Sub-theme 1: Majority of the participants have negative attitudes towards HIV Counselling and Testing services

The participants expressed feelings of fear to know their status, humiliation, ignorance and being afraid of embarrassment to partners. One of the participants even said:

‘If my wife go to HCT and found to be negative I am therefore negative.’ (Participant 15)

Initially all the participants (*n* = 15) indicated that they did not want to be tested: during the interviews they reflected dissatisfaction.

#### Sub-theme 2: Men are afraid of being tested positive

The majority (*n* = 11) of the participants indicated that they were afraid to be tested, and revealed that to know their status would be detrimental to their aftermath survival. The following are some of the sentiments of the participants:

‘I fear that I will die soon if am found positive, because most of us men we are not faithful to our partners, and men do not want to be responsible for medication.’ (Participants 2 & 15)

One of the participants revealed that if found to be positive his wife, kids, and the public would look down upon him and his wife could leave him.

#### Sub-theme 3: Respondents accuse the behaviour of counsellors as a catalyst of negative attitude towards HIV Counselling and Testing services

Twelve of the participants’ echoed that they did not have trust in the behaviour of lay counsellors who were providing HCT services; hence, they developed negative attitudes towards HIV testing and counselling. One of the participants confirmed that:

‘The behaviour of counsellors and those who test they do not have professional secrecy, we don’t like them because they don’t seem to keep confidential information, as a men I prefer to be treated by a private doctors.’ (Participant 14)

This response points to the idea that the majority of men in Vhembe have a feeling of dislike towards HCT service; the reason being the perceived ethical conduct of HCT service providers.

### Theme 3: The socio-cultural factors that prevent the participants from going for HIV Counselling and Testing services

#### Sub-theme 1: Men believe in cultural practices rather than the medical and professional practices

Thirteen out of 15 respondents expressed varied ideas on the aspect of cultural practices such as circumcision and traditional treatment by traditional healers as they agreed that it was one of the factors that men prefer to HCT services. To emphasise issues related to these participants simultaneously said:

‘Men they don’t believe in testing and do not believe that AIDS can be treated by western medicine but believe in traditional medicine.’ (Participants 3, 4, 11, 12, 1, 3 & 10)

These verbalised above statements support the notion that in cultural beliefs among different social settings among Vhembe men, an ingredient of reluctance toward utilising HCT services has emerged.

The study findings revealed that most of the men believed in cultural practices like circumcision and that there is no need to get HCT services. This is what most respondents (*n* = 13) concurred on:

‘I think when you are circumcised you don’t need to visit the HCT center because our ancestors and forefathers had been living without all these mushrooming services like HCT and many more like FPD …my view is men especially of older age still stick to their traditions you see!’ (Participants 1, 2, 3, 4, 5, 6, 11, 12, 15 & 7)

### Theme 4: Political, administrative and legislative factors that prevent participants from utilising HIV Counselling and Testing services

#### Sub-theme 1: Men expressed that politics is failing to relay and cooperate the medical and traditional health practitioners for a common goal

Six of 15 participants have expressed the existence of political barrier factors making them reluctant to attend HCT services in primary health facilities. One of the participants confirmed by saying:

‘I think most men are still glued to their traditional treatment and practices like traditional healing and circumcision; however it is the duty of the ruling party, through responsible departments to give enough information to these people to change their beliefs or to amalgamate the two.’ (Participant 9)

#### Sub-theme 2: Men view administrative personnel and labour laws as hindrances to HIV Counselling and Testing service utilisation

All participants (*n* = 15) have expressed the influence of poor administration structure in turning away the attitudes of men. Corruption purported by media and rumours, which include the alleged embezzlement of funds, make men, thinks that HCT services are incompetent thus makes them not want to go there. The allegations regarding the selling of ARVs drugs by staff in several facilities also contribute to poor administrative role and corruption stated by participants:

‘One of the participants said ‘I think most of us we believe in doctors, but when it comes to these counselors, they seem to be lay people, and some of them we had they even sell ARVs at their places and this is corruption, most don’t like that.’ (Participant 1)

The implication is that men will resist accessing HCT knowing that the government at the end of the day will give them treatment if they happen to be ill. The majority (*n* = 14) of the participants echoed the same sentiment pertaining to governmental policies they confirmed that:

‘Every month people are paying for medical insurance so they feel assured after they feel sick.’ (Participants 3, 4, 8, 6, 11, 12, 14, 13, 15, 1, 2 & 10)

### Theme 5: Gender and stigma-related factors that influence participants’ level of utilisation of HIV Counselling and Testing services

#### Sub-theme 1: Gender and stigma-related issues have made many men reluctant to utilise HIV Counselling and Testing services

All of the 15 participants verbalised that gender related issues have been central to the majority of men to choose not to visit the HCT. One of the participants related that men don’t want to be tested by women and said:

‘Men are afraid that if they are tested positive, they will be rejected by the community, sometimes the people who conduct testing are usually women and men don’t feel comfortable to see women knowing their health statuses.’ (Participant 7)

#### Sub-theme 2: Participants identified ways of enabling men to utilise HIV Counselling and Testing services

All the participants identified many strategies that can be adopted to ensure the utilisation of HCT services by men particularly in Vhembe district and SA in general. They identified educational awareness through campaigns and mass involvement of all departments in a bid to change the attitudes of men towards HCT services. Some of the sentiments of the participants were:

‘Health talks to be given using all kinds of media, even in traditional meetings (Khoroni), schools, public places like taverns, beer halls, and sheebens and in political parties’ gatherings.’ (Participants 8, 3, 5, 7, 6, 1 & 10)

## Discussion

In the present study, it was observed that most of the interviewees were aware of HCT services. Moreover, they confirmed that HCT services were offered in Vhembe schools, work places, private clinics, hospitals, and by private doctors. Stigma such as human reactions to disease and attitudinal factors have been central to lower numbers of men seeking counselling and testing from primary health facilities. Participants blamed politicians because of their role in lobbying governmental health policies which are not promoting men to seek HCT services.

The above mentioned results of the study were consistent with a study which was conducted in Ethiopia which revealed highest level of HCT knowledge and awareness while a small percentage revealed lack of awareness.^1^ It is evident that awareness campaigns, which were conducted in SA like Khomanani, Love life, and Soul city, were successful and showed impact to the lives of communities. Khomanani was the least successful campaign and its effectiveness has increased by more than 50.0% between 2005 and 2008.^2^ A study conducted by Perry et al. revealed the availability of HCT; the study showed that 64.4% of males agreed with the availability of HCT in local centres but further indicated that availability of free Voluntary Counselling and Testing (VCT) services for HIV among rural migrants is lacking.^4^

The findings of the present study revealed that many men in rural Vhembe lack the proper knowledge and the essence of HCT, giving reasons of poor information reaching the rural population. Consistently, in a study conducted by Tesfaye, Ingvild, and Knut in Ethiopia on factors affecting voluntary HCT among men in Ethiopia, the results showed that 96% of men reported that they had heard of HIV, of these, 90.2% of men responded that they knew how to prevent or avoid HIV infection.^1^ The level of knowledge was higher among urban than rural men as measured by parameters, and the results showed marked differences in HIV and AIDS related stigmatising beliefs as well as in the utilisation of VCT services among men.

The study by Khan et al. concurred the idea that although information about HCT services is being conveyed, it is being twisted on the way to evangelise the intervention of HCT to the people in the different communities.^4^ Going a mile with same sentiment, it might be out of beliefs purported by lack of knowledge that make people fail to understand the scope and mission of HCT. The results of the present study differ with the findings of the study conducted by Wagenaar on factors affecting VCT where 47.0% of the participants noted the benefit of the test and remarked the importance of and relief associated with knowing one’s HIV status and the ability to live a better life or live positively after testing, including protecting oneself and partners.

The present study revealed the negative perception of the participants about HCT as some verbalised that they feel like they are going to die anyway and do not think it is necessary to know one‘s status. In the same study, the majority revealed that they are afraid to feel pain and cope with knowledge that they are HIV positive. Consistently, a study on voluntary counselling and testing among men in rural western Uganda confirmed that men were worried about taking an HIV test because to them having a positive result meant a myth of imminent death.^5^

The study conducted by Bwambale et al. on voluntary HCT among men in rural western Uganda, consistently showed that the majority of men receive information about HCT through the health workers (75.2%) and this contradicts findings of the study where men viewed the code of conduct for counsellors infringing on the clients’ emotions, which in turn changed their attitude towards HCT services.^10^

The men from Vhembe have blamed political and administrative influences in failing to unite the medical and traditional practitioners for the common goal. The above statement indicates that the failure of political structures to strike a balance of cooperation between the lay healers and the professionals to counter the HIV pandemic thus increases the rate of men utilising the modernised HCT services. In this study, there was a strong feeling that politics is playing a barrier role in increasing reluctance for men to access HCT services. According to UNAIDS the importance of involving and mobilising a range of stakeholders in dealing with the epidemic, because they played a number of different roles, cannot be over-emphasised. It means making serious efforts and maintaining both formal and informal relationships within and between government, communities, business, and civil society.^1^

There may be negative implications on HCT if DOH is not working closely with traditional health practitioners in the District because most of the men do attend traditional practitioners when they are sick. The positive implications are that a referral system can easily be implemented between medical doctors and traditional health practitioners.

Kloos purported that stigma and discrimination have taken their toll in Ethiopia not only at the work place, in housing, health facilities, schools, and family and personal relations; but also in medical services, discouraging people from being tested for HIV.^11^ A review study conducted in SA showed that HIV and/or AIDS related stigma drives the pandemic out of the public view and is reducing both individual and societal efforts for behaviour change.^11^ Stigma as a human reaction to disease has been central to lower numbers of individuals turning up to seek counselling and testing; furthermore it was found to be exacerbated by different sexes occupying counselling and testing offices. Snow et al. consistently supported this by defining HIV and/or AIDS stigma as prejudice, discounting, discrediting, and discrimination directed at people perceived to have HIV and/or AIDS.^2^

This points out the idea that although the majority men are aware of HCT services in Vhembe, it seems that their attitude is the fear of being stigmatised after being tested positive or just being perceived as being a victim. The stigmatisation is being perceived as emanating from the community members as well as from the counsellors, who are said to usually be women. The study also showed that HIV and AIDS related stigma is a well-documented barrier towards the uptake of HIV and AIDS related services including HCT and ART.

Fear of stigma and discrimination as registered by many participants deter men from testing and consulting at public health facilities. The notion and response of men in fearing women at the testing sites was also supported by a study conducted at Sekhukhune District in 2011, where most of the men and other groups appear to be reluctant to use services where local community members are employed for fear that the confidentiality of their status would be compromised.^5^

### Recommendations

The following recommendations were made.

The myth regarding the HCT programme that it fuels the spread of HIV can be corrected by proper methods of health information dissemination as well as combining the medical and traditional practitioners together for the common goal of the HCT mandate.

Establishment of a traditional and medical health practitioners’ forum in the district must be strengthened to enforce relations and proper coordination of HCT services.

Provincial HIV and AIDS programme managers should also make efforts to involve men as partners in the fight against HIV and AIDS. Provincial men’s sector needs to be strengthened and capacitated by programme managers.

When strategic plans for National Strategic plan (NSP) on HIV and/or AIDS, STIs, and TB are reviewed, it is necessary that men’s sectors across the provinces are involved so that they make input on the running of HCT services in the county.

To increase the knowledge of HCT, the national department must continue with the national campaigns like Soul city and love life so that information on HCT is cascaded to all men. Politicians including the Minister of Health and MEC should lead the AIDS structures and ensure that they include educational programmes that will encourage men to access HCT services. In all strategic meetings, all politicians need to encourage men to go for HCT.

### Developing attitudinal approach in counselling

The study recommended that the positive attitudinal approach should be entwined in the counsellor’s training.

#### Personal worth

An attitude of personal worth is the viewpoint the counsellor holds about self and others. It involves acknowledging and accepting cultural differences, recognising the autonomous rights of others, showing respect for humanity, and acknowledging the client as an individual.

#### Personal integrity

An attitude of personal integrity means that the counsellor nurse maintains honesty towards herself in her dealings with herself and with others.

#### Advocacy

Advocacy is an attitude of support that safeguards the rights and integrity of the patient. It includes standing up or fighting for the good of the client.

This study has managed to highlight various factors that contribute to men’s reluctance to seek HCT at primary health care facilities. However, there is still need for further research to determine whether there are differences in the factors hampering men seeking HCT services in rural and urban areas.

## Conclusion

There was a high level of HCT awareness; however its utilisation was being lowered by an array of factors. The conclusions point to political, administrative, and attitudinal barriers; stigma; and cultural practices like circumcision as some of the factors leading to low level of utilisation of HCT.
